# Screening for Volatile α-Unsaturated Ester-Producing Yeasts from the Feces of Wild Animals in South Africa

**DOI:** 10.3390/life12121999

**Published:** 2022-11-30

**Authors:** Mélissa Tan, Yanis Caro, Juliana Lebeau, Alain Shum-Cheong-Sing, Jean Marie François, Thierry Regnier, Thomas Petit

**Affiliations:** 1 Laboratoire de Chimie et Biotechnologies des Produits Naturels, Université de la Réunion, 97400 Reunion, France; 2 Département Hygiène, Sécurité et Environnement (HSE), IUT de la Réunion, 97410 Reunion, France; 3 Toulouse Biotechnology Institute (TBI), INSA Toulouse, 31400 Toulouse, France; 4 Department of Biotechnology and Food Technology, Tshwane University of Technology, Pretoria 0001, South Africa

**Keywords:** natural flavors, Ehrlich’s metabolic pathway, α-unsaturated esters, branched-chain amino acids, *Saprochaete suaveolens*, non-conventional yeasts, feces of wild animals, microbial biodiversity

## Abstract

α-unsaturated esters are fruity-aromatic compounds which are largely spread in the volatilome of many different fruits, but they are rarely found in the volatilome of yeasts. The yeast *S. suaveolens* has been recently shown to produce relatively high amounts of α-unsaturated esters and it appears to be an interesting model for the production of these compounds. This study aimed to isolate new α-unsaturated ester-producing yeasts by focusing on strains displaying a similar metabolism to *S. suaveolens*. While the production of α-unsaturated esters by *S. suaveolens* is believed to be closely related to its ability to grow on media containing branched-chain amino acids (isoleucine, leucine and valine) as the sole carbon source (ILV^+^ phenotype), in this study, an original screening method was developed that selects for yeast strains displaying ILV^+^ phenotypes and is able to produce α-unsaturated esters. Among the 119 yeast strains isolated from the feces of 42 different South African wild animal species, 43 isolates showed the ILV^+^ phenotype, among which 12 strains were able to produce α-unsaturated esters. Two interesting α-unsaturated esters were detected in two freshly isolated strains, both identified as *Galactomyces candidus*. These new esters were detected neither in the volatilome of the reference strain *S. suaveolens*, nor in any other yeast species previously studied for their aroma production. This work demonstrated the efficiency of an original method to rapidly screen for α-unsaturated ester-producing yeasts. In addition, it demonstrated that wild animal feces are interesting resources to isolate novel strains producing compounds with original aromas.

## 1. Introduction

Food flavors are the result of a complex mixture of volatile organic compounds (VOCs) whose quantitative and/or qualitative changes can influence consumers’ overall flavor perception [1]. As recreating a specific food flavor can be a difficult task, using flavoring agents could be a good alternative. Nowadays, such flavoring agents are mainly produced *via* chemical synthesis, which leads to “synthetic flavors”. Alternatively, “natural label” flavors are produced through extraction from plants or micro-organisms. Synthetic flavors still account for almost 80% of the global food market as they lead to low-cost products due to high production efficiency. However, consumer interest in artificially flavored food products has steadily declined in recent decades, encouraging the search for new methods to produce natural flavors. Extraction from plants was the first route explored, but it is highly dependent on seasonality constraints and generally shows low production yields. In addition, the growing demand for raw materials generates increasing environmental challenges [2,3,4].

As a consequence, research is directed towards the production of natural flavors using biotechnological approaches that allow the production of natural flavor compounds that are stable and compatible with industrial processes with as limited an environmental impact as possible [3,4,5]. Among them, the production of aromas using micro-organisms offers a wide range of possibilities due to the great diversity of their metabolisms allowing them to form various complex VOCs. Among the micro-organisms, yeasts, particularly those derived from the *Saccharomyces* spp. genera, have been widely described as good producers of aromas, notably in the form of alcohols, esters, ketones, aldehydes and acids from amino acid and fatty acid synthesis and degradation [6,7]. Additionally, numerous studies have shown that yeasts from other genera, also known as non-conventional yeasts (NCY), can play an important role in the aromas of many complex fermented drinks such as wine, beer or kefir [8,9,10,11,12,13]. Similarly, many NCYs have been studied for their production of VOCs on complex media such as yeast–peptone–dextrose (YPD) medium [12,14,15,16,17,18,19,20]. With more than 30 different VOCs, including esters detected in their headspace, *Pichia kluyverii*, *Torulaspora delbrueckii* and *Saprochaete suaveolens* (former *Geotrichum fragrans*) have been shown to be promising yeast strains for both flavor production and application in the natural flavoring of food products such as beer [3,9,12,16,17,21,22,23,24,25,26,27].

*Saprochaete suaveolens* has received particular attention as it has been shown to produce highly aromatic α-unsaturated esters such as ethyl but-2-enoate (ethyl crotonate). The volatilome of *S. suaveolens* is particularly known to produce α-unsaturated esters under the form of tiglates such as ethyl (E)-2-methylbut-2-enoate (ethyl tiglate), 2-methylpropyl (E)-2-methylbut-2-enoate (isobutyl tiglate), 3-methylbutyl (E)-2-methylbut-2-enoate (isoamyl tiglate), butyl (E)-2-methylbut-2-enoate (butyl tiglate) and propyl (E)-2-methylbut-2-enoate (propyl tiglate). They are all known to generate strong fruity-like aromas. Even if these compounds are largely spread in the volatilome of many different fruits such as mango, siriguela, cocoa, quince and apple [28,29,30,31,32], only a small number of yeasts, belonging mainly to the *Galactomyces*, *Geotrichum* and *Saprochaete* genera, were shown to produce α-unsaturated esters, with *S. suaveolens* producing the largest variety of those specific esters [17,19].

Based on this finding, *S. suaveolens* was identified as a good model for the production of α-unsaturated esters in yeasts, thus motivating elucidation of the metabolic pathway of ester production in this yeast. It has thus been demonstrated that in *S. suaveolens*, α-unsaturated esters are produced through the catabolism of branched-chain amino acids (BCAAs, isoleucine, valine and leucine) *via* the β-oxidation pathway [33]. This pathway involves an enoyl-CoA intermediate, which can be esterified into α-unsaturated esters by an alcohol acetyltransferase or which can continue its catabolism *via* the fatty-acid β-oxidation pathway. This second possibility results in the formation of acetyl-CoA units, which are then incorporated into the TCA cycle and further into the glyoxylic shunt, allowing *S. suaveolens* to grow on media containing only BCAAs as the sole carbon source. Moreover, more recently, it has been demonstrated that for *S. suaveolens*, the loss of this ability could be attained *via* inactivation of the enoyl-CoA hydratase of the β-oxidation pathway, which results in an enhancement in total VOC production, including α-unsaturated esters [34]. Based on this observation, the assumption was made that the ability to grow on media containing only BCAAs as the sole carbon source (ILV^+^ strain) is closely related to the formation of α-unsaturated esters and could, therefore, be a useful way to select for new interesting ester-producing yeast strains from new ecological niches such as wild animal feces.

Indeed, currently, research is focusing on the need to explore new ecological niches to isolate yeast strains. Among these niches, animal feces have attracted interest as they contains yeast strains with particular properties and useful applications. For instance, Lorliam et al. (2013) discovered various yeasts of different genera including *Geotrichum*, *Candida*, *Sporopachydermia*, etc. in a study of the biodiversity of yeast species in buffalo dung from Thailand [35]. In India, Ganesh-Kumar et al. (2010) demonstrated the presence of various anti-microbial fungi (anti-*Candida albicans*, anti-*Micrococcus luteus*, etc.) in the feces of elephants, rhinoceroses and tigers [36]. This tends to suggest that wild animal feces might be a suitable source to explore for the discovery of new strains producing natural fragrances. South Africa is well known for its rich ecosystems and its natural parks, game reserves and animal rehabilitation centers. Thus, it constitutes an interesting and unexplored area to collect excrement from wild animals, which could provide new particular yeasts.

Based on the newly identified pathway of esters in *S. suaveolens*, in this study, an original method to isolate new yeast strains producing α-unsaturated esters from wild animal feces in South Africa was developed. Several campaigns to collect feces from various wild animals in South Africa were performed before isolating yeast strains on yeast–peptone–dextrose medium, supplemented with 0.1 g·L^−1^ of chloramphenicol (YPD-chloramphenicol). Then, a screening method based on the capacity for rapid growth of the cells on media containing BCAAs as the sole carbon source was implemented. Finally, the ability of the ILV^+^ strains to produce α-unsaturated esters and their chemical characteristics during growth on YPD medium, using headspace solid-phase micro extraction (HS-SPME) coupled to gas chromatography and mass spectrometry analysis (GC/MS), were investigated.

## 2. Materials and Methods

### 2.1. Biological Materials

Wild-type *Saccharomyces cerevisiae* CEN.PK 112-2N [37] and *Saprochaete suaveolens* (former *Geotrichum fragrans*) strains previously isolated from Pitaya fruits (dragon fruit, *Hylocereus polyrhizus*) in Reunion Island, France [16] were used as reference strains. Fresh feces of wild animals were collected aseptically from different locations in zoos, game reserves and national parks of South Africa (Table 1). For each location, official authorization was obtained from local authorities. Collection was performed using pre-labeled sterile swabs or tubes, which were hermetically sealed and transported from the site to the laboratory. Then, samples were kept at 4 °C until use. Additionally, freeze-dried samples of the wild animals’ feces, collected aseptically, were obtained from anonymous donors. Overall, 42 different species of animals were investigated and 62 feces samples were collected. All feces samples and their origins are presented in Table 2.

### 2.2. Isolation and Selection of ILV^+^ Yeast Strains

For swab samples, sterile swabs were inserted into the dung, and then, added directly to 10 mL of sterile Ringer’s solution. For other solid samples, 1 g of feces was weighed beforehand and dissolved in Ringer’s solution. Serial dilutions were then prepared and 100 µL of each dilution was spread on the surface of yeast–peptone–dextrose agar medium (Biolab diagnostics LTD, South Africa) supplemented with 0.1 g·L^−1^ of chloramphenicol (EMDQ Millipore Corp. Billerica, MA, USA) (YPD-chloramphenicol) to promote the growth and selection of yeast cells. Inoculated Petri dishes were then incubated at 30 °C for 48 h until cell colonies were completely formed. Colonies were then isolated by repeating subcultures on YPD-chloramphenicol agar medium, and then, stored at 4 °C. For other samples, 1 g of lyophilized feces was suspended and vortexed into 10 mL of sterile nutrient broth (Merck, South Africa) prior to incubation at 25 °C for 5 days before plating on YPD-chloramphenicol medium.

The selection of strains displaying the ability to grow on media containing only BCAAs as the sole source of carbon (ILV^+^ strains) was carried out as follows: 5 mL of YPD broth medium was inoculated with each isolated strain and the culture was incubated overnight at 30 °C under gentle shaking (230 rpm). After centrifugation (13,000 rpm, 5 min), the microbial biomass was washed twice with 5 mL of sterile physiological water and suspended in 10 mL of sterile physiological water. Cell content in the suspension was determined using a Thoma cell-counting chamber. A suspension of cells at 10^6^ cells·mL^−1^ was then prepared in sterile physiological water, which was then diluted to 10^5^ and 10^4^ cells·mL^−1^ *via* serial decimal dilution. Each cell suspension (5 µL) was dropped twice onto yeast nitrogen-based medium plates supplemented with 1 g·L^−1^ of isoleucine, leucine or valine or 2 g·L^−1^ of glucose (YNB-Ile, YNB-Leu, YNB-Val and YNB-Glc, respectively). The reference *S. suaveolens* and *S. cerevisiae* strains were prepared following the same protocol and were used as positive and negative controls, respectively. Petri dishes were then incubated for 48 h at 30 °C before analysis.

### 2.3. Qualitative and Semi-Quantitative Analysis of Volatile Organic Compounds (VOCs)

Qualitative and semi-quantitative analysis of volatile organic compounds (VOCs) produced by yeast were performed during growth on yeast–peptone–dextrose (YPD) medium. Qualitative analysis was managed for all ILV^+^ yeast strains to search for isolates that produce interesting α-unsaturated esters. Then, semi-quantitative analyses were performed only on selected strains to compare their overall VOC production with that of *S*. *suaveolens*. YPD medium was chosen according to the literature because it is rich enough to allow conventional or non-conventional yeasts to produce a large panel of aroma compounds [3,11,13,14,15,17,18,19,37]. Strains were inoculated into a 20 mL screw tube containing 15 mL of inclined YPD-agar slant and incubated at 30 °C for 24 h. Then, the vials were sealed and re-incubated for 24 h at 30 °C. For qualitative analysis, the headspace of inclined cultures was directly subjected to headspace solid-phase microextraction (HS-SPME) analysis using a 2 cm long fiber coated with 50/30 µm divinylbenzene/Carboxen on polydimethylsiloxane bonded to a flexible fused silica core (Supelco Inc, Bellefonte, PA, USA). For semi-quantitative analysis, prior to microextraction, 10 µL of octan-1-ol (at 0.5 g·L^−1^ in dichloromethane) was added into the sealed vials containing 15 mL of inclined YPD-agar slant as an internal standard. The fiber was then exposed to the headspace of the inclined cultures for 15 min at 30 °C and inserted into the injection port at 250 °C for 2 min. Metabolites were separated *via* gas chromatography (GC/MS) using a ZB-5MSI column (30 m × 0.32 mm × 0.25 µm film thickness) coupled to a mass spectrometer (Shimadzu GCMS-QP2010 Ultra, Shimadzu, Kyoto, Japan). The carrier gas (H_2_) was set at a flow rate of 1.4 mL·min^−1^. The column temperature was maintained at 45 °C for 2 min and raised at 4 °C·min^−1^ up to 230 °C. This temperature was finally maintained for 5 min to desorb as much of the compounds as possible. Volatile organic compounds were identified by comparing their mass spectra with the NIST database (www.chemdata.nist.gov (accessed on 14 November 2022)) and their experimental incidence rate ratios (IRR_exp_, Kovats index) with the theorical incidence rate ratios (IRR_th_) found in the literature. For the semi-quantitative analysis, the concentration of each compound was determined by comparing the area of the peak of the compound with the area of the internal standard (octan-1-ol at 0.5 g·L^−1^).

### 2.4. Colony PCR and Yeast Identification Using rDNA Sequence Analysis

Identification of the yeast strains was performed at the molecular level *via* amplification and sequencing of the ribosomal RNA-encoding DNA (rDNA) Internal Transcribed Spacer (ITS) region consisting of the 5.8S rRNA gene and two flanking regions (ITS1 and ITS2). The species-specific polymorphism of the ITS region of yeast rDNA is a tool commonly used as a reliable way in distinguishing yeast species [38].

Colony PCR was carried out using fresh cells as an amplifiable template. Cells were directly collected from a fresh yeast colony using a 1 µL sterile loop. Cells were suspended in a 100 μL PCR reaction mixture containing 0.5 µM primer ITS1 (5′ TCCGTAGGTGAACCTGCGG 3′), 0.5 µM primer ITS4 (5′ TCCTCCGCTTATTGATATGC 3′), 200 µM of each deoxynucleotide, 1× buffer and 2.5 units of DNA polymerase (Qiagen, Hilden, Germany). The PCR conditions were as follows: initial denaturation at 95 °C for 5 min; 35 cycles of denaturing at 94 °C for 1 min, annealing at 55.5 °C for 1 min and extension at 72 °C for 1 min; and a final extension at 72 °C for 10 min. PCR products were analyzed *via* electrophoresis on 2% agarose gels, with 1× TAE buffer at 100 V for 20 min. Gels were stained with Midori Green (Nippon Genetics Europe, Düren, Germany) and visualized under UV light. The size of the amplified DNA fragments was estimated by comparing them against a 1 kb DNA ladder (1 kbp ladder, O’GeneRuler, ThermoFischer, Waltham, MA, USA). Sequencing of the PCR products was performed by Eurofins Genomics (Germany). Sequences were analyzed and compared to reference data available at the National Center for Biotechnology Information (NCBI; http://www.ncbi.nlm.nih.gov/blast (accessed on 14 November 2022)) using BLAST (Basic Logarithmic Alignment Tool). Yeast identification was assumed if the query sequence showed >99% identity with DNA sequences deposited in the NCBI database. Sequence alignments with reference sequences from the MycoBank database were also performed to support the results obtained. The obtained ITS sequences of strains S12 and S91 were deposited to the GenBank database and assigned accession numbers of “OM397075” and “OM397076”, respectively.

### 2.5. Statistical Analysis

Experimental data were subjected to factor analysis (principal component analysis method) and cluster analysis (Ward’s method) using XLStat Applied Sensory software (2020.1.3).

## 3. Results

### 3.1. Mammalian Feces: An Original Yeast Species Resource

Data from the literature suggest that animals’ gut microbiomes are strongly dependent on the host phylogeny [39,40,41]. Therefore, a fecal microbial population of 42 different wild animal species was investigated. The animal species were distributed as follows: 13 carnivorous (23 samples) and 16 herbivorous mammals (25 samples), 8 raptors (10 samples), 3 birds (3 samples) and 1 reptile (1 sample) (Table 2). However, diet and habitat also have a strong effect on the microbial diversity of an animal’s gut [39,41]. Thus, to distinguish the possible effects of animal domestication on microbial populations in the feces of wild animals, samples of feces coming from domestic and wild locations, including zoos, game reserves and national parks in South Africa, were included (Table 1). However, due to the constraints associated with collecting samples from wild habitats (especially for carnivorous mammals), feces collected from animals living in these habitats were less numerous than those collected from domestic habitats (14 wild against 37 domestic habitats, respectively). Moreover, the origin of the 11 freeze-dried samples offered by an anonymous donor was not specified. Microbial strains from these 62 samples were isolated on YPD-chloramphenicol medium in order to prevent the development of bacteria and to promote yeast selection. With this method, 119 different yeast isolates were obtained, with 89.9% coming from mammalian feces (40.3% from herbivores and 49.6% from carnivores), and 10.1% from raptors (4.2%), birds (2.5%) and reptiles (3.4%).

All the feces collected from mammals (herbivorous or carnivorous) allowed for the isolation of at least one yeast species on YPD-chloramphenicol medium, while feces from others families did not. For example, among the seven species of raptors, only the dropping of the white-headed vulture (*Trigonoceps occipitalis*) and the tawny eagle (*Aquila rapax*) made it possible to isolate a yeast species. Similarly, out of the three species of bird investigated, one species, namely the Abyssinian hornbill (*Bucorvus abyssinicus*), did not display any yeast strains in this sample (Table 2). Thus, the distribution of the yeast species in the feces of wild animals was not uniform. This result is in good agreement with the theory of Hammer et al. (2019), which showed that three groups of animals could be distinguished according to their microbiota [40]. The first group contained animal species for which the microbiome is essential and abundant, while the second group contained animal species for which the microbiome is not essential and very weak. In both cases, the microbiome can be composed of micro-organisms (bacteria, archaea, viruses, fungi or protozoa) or helminths, with different proportions according to the animal species [42]. Finally, the third group contains animal species for which a microbiome is absent because their presence is deleterious. Thus, according to the results obtained in this study, raptors and birds seem to belong to the third group; this is consistent with their gastric high acidity, which prevents the yeast from developing in their organisms [40,43,44]. Thus, the feces of these kinds of animals cannot be considered good candidates to screen for new yeast species.

Conversely, mammalian animals seem to belong to the first group and benefit from symbiosis with a microbiome. However, only feces from lion *(Panthera leo*, Emoya big cat sanctuary), leopard *(Panthera pardusi,* Lory Park) and springbok *(Antidorcas marsupialis*, Moreleta kloof nature game reserve) displayed more than three physiologically different strains. This shows that even if fecal samples are known to display high bacterial populations, the yeast biodiversity is probably very low. However, it cannot be excluded that the chloramphenicol added to the medium to reduce bacterial development during the screening might have prevented the growth of some sensitive yeast species. It is also possible that there were missing auxotrophic compounds in the culture medium used for the selection of the strains, which may have prevented the growth of these selective strains.

Other lion, leopard and springbok fecal samples showed much lower numbers of yeast species. Thus, the habitat seems to play an important role in the microbiota of mammals within specific species. This was particularly noteworthy for the feces of elephants *(Loxodonta africana)* living in natural habitats such as Kruger National Park, for which no strain was isolated, while three different strains were isolated from the droppings of elephants living in the Elephant Sanctuary (Table 2). In accordance with other studies [41,44,45,46], animal nutrition has also played an important role in the diversity of their microbiota, especially within domestic habitats. Indeed, 78.6% of fecal samples from animals living at Lory Park Zoo contained at least one yeast species, whilst only 33.3% of fecal samples from animals living in Moholoholo Animal Rehabilitation Center displayed one yeast species. However, it is necessary to highlight that Moholoholo Animal Rehabilitation Center aims to treat injured wild animals before releasing them back into nature (www.moholoholo.co.za (accessed on 14 November 2022)).

The current study, therefore, demonstrates that the excrements of mammalian animals originating from natural or domestic habitats represent an interesting source of yeast species. However, to maximize the chance of isolating new strains, domestic habitats involving interventions in their diets by humans that can alter the development of micro-organisms should be avoided. Moreover, the feces of mammalians can provide the yeasts with an original metabolism. Indeed, the metabolism of conventional yeasts such as *Saccharomyces cerevisiae* does not allow its development on media containing only BCAAs as a carbon source (ILV^+^ phenotype), even if this phenotype has been described for certain filamentous fungi and yeast strains [47,47,48]. In this study, 36% of the yeast species (43 out of the 119 isolated yeasts) showed the ILV^+^ phenotype (Table 2). With the exception of strain S88, which was isolated from the droppings of a Nile crocodile (Lory Park Zoo), all the strains came from the feces of herbivorous and mammalians, suggesting that diet does not play an important role in the development of the ILV^+^ yeast strain in animal feces. In *S. suaveolens*, the ILV^+^ phenotype is due to the catabolism of BCAAs *via* the β-oxidation pathway. This catabolism results in the formation of acetyl-CoA units, which are then incorporated into the TCA cycle and further into the glyoxylic shunt [33]. The specific biological role of this pathway is not yet elucidated, but it allows the strain to develop in the absence of another assimilable carbon source. With the surprising high amount of the ILV^+^ strain isolated in our study, the assumption can be made that it is the result of an adaptation to media containing relatively little assimilable carbon, such as feces.

### 3.2. Selection of ILV^+^ Strains Producing Volatile Organic Compounds (VOCs)

Based on the hypothesis that growth on BCAAs could be accompanied by high α-unsaturated ester production [34], the volatilome, i.e., the volatile organic compounds produced by the 43 ILV^+^ isolated yeasts and of *S. suaveolens* (S0), used as reference, was qualitatively examined using headspace-solid-phase micro-extraction followed by gas chromatography coupled with a mass spectrometry detector (HS-SPME-GC/MS) after 48 h of growth on YPD medium at 30 °C. Among the 43 ILV^+^ isolates, 21, namely S4, S17, S22, S28, S33, S40, S44, S58, S60, S62, S63, S65, S72, S78, S92, S97, S117, S121, S123, S124 and S125, did not produce any VOCs. On the contrary, 50 different VOCs were detected in the volatilome of the 22 remaining isolates and in the control *S. suaveolens* strain. These VOCs were classified into four main categories, namely acids, alcohols, esters and α-unsaturated esters, and are presented in Table 3.

### 3.3. VOCs Produced by ILV^+^ Aroma-Producing Isolates

As indicated above, 21 out of the 43 ILV^+^ yeast species isolated from feces were unable to produce any VOCs, suggesting that they strictly assimilated the BCAAs through the β-oxidation pathway for growth [33]. On the other hand, the 22 ILV^+^ flavor-producing strains and the positive control *S. suaveolens* (S0) strain produced a large panel of VOCs, with esters being the most predominant (Table 3). According to the KEGG pathway database (www.genome.jp), the major metabolic pathways involved in the production of these VOCs included the fatty-acid β-oxidation (ethyl hexanoate, ethyl octanoate, etc.), butanoate (butyl acetate, butyl butanoate, ethyl butanoate, etc.), propanoate (butyl propanoate) and pentanoate (ethyl pentanoate) pathways.

A single type of alcohol, namely 2-phenylethanol, was detected in the headspace of *S. suaveolens* (S0) and 13 of the 22 investigated strains (Table 3 and Table 4). This result may seem surprising because yeasts are well-known producers of alcohol compounds (notably ethanol) during growth [7,14,15,54] and because the production of higher alcohols such as 2-methylbutanol and 3-methylbutanol by *S. suaveolens* has already been observed in other studies [17,33,55,56]. However, the analytical method used in this study is not sensitive and resolutive enough to allow the visualization of these compounds on the resulting chromatogram.

Additionally, acid production was not widespread among the strains studied. Indeed, only three different acids produced by only four different isolates were found (Table 3 and Table 4). The most frequently detected acid was 3-methylbutanoic acid (sweet taste), which was produced by the S37, S87 and S107 isolates. This compound is generated from the catabolism of leucine through the Ehrlich pathway and is rarely detected in the volatilome of yeasts because it is usually rapidly esterified to its corresponding ethyl ester (ethyl 3-methylbutanoate). However, 3-methylbutanoic acid has already been detected in some yeast strains such as *Pichia guillermondii*, *Pichia kluyveri* and *Sporidiobolus pararoseus* [12,16].

As already reported for the *S. suaveolens* species [16,17,33,56], ester compounds were the most predominant (i.e., 37 to 50) in the volatilome of the isolated yeast species, with the exception of the S74 and S82 strains, which only produced 2-phenylethanol (Table 3 and Table 4). The S0 (*S. suaveolens*), S12 and S91 strains displayed the largest diversity of esters in their volatilome. In particular, the volatilome of S0 contained about 30 different compounds in this category, and of them, 13 were not identified in the other strains. However, pentyl butanoate, which harbors an apricot/pineapple-like flavor and which is frequently used in food and perfumery, was not detected in the volatilome of S0, but was detected in the volatilome of the S12 and S91 strains. This result is interesting because this compound is mostly found in fruits (apple and cocoa bean) but is rarely detected in the volatilome of micro-organisms. The high distribution of esters in the volatilome of the isolates suggested the occurrence of several efficient esterase activities that ensure the transformation of alcohols and acids (resulting from the EP). Hence, one might expect that the assimilation of BCAAs in the β-oxidation pathway should allow the production of α-unsaturated esters *via* the esterification of enoyl-CoA, resulting the partial β-oxidation of amino acids [33]. However, the results show that 10 out of the 22 ILV^+^ isolated yeasts did not produce α-unsaturated esters. This results indicate that the capacity of aroma-producing yeasts to assimilate BCAAs in the β-oxidation pathway is not necessarily linked with the production of α-unsaturated esters.

From the 12 α-unsaturated ester-producing strains, five α-unsaturated esters were produced by both S0 and the strains isolated in South Africa, with ethyl (E)-2-methylbut-2-enoate (ethyl tiglate) and ethyl (E)-3-methylbut-2-enoate (ethyl 3-methylcrotonate), respectively, produced *via* the β-oxidation of isoleucine, and leucine being the most distributed within the isolates. Butyl (E)-2-methylbut-2-enoate and propyl (E)-2-methylbut-2-enoate (butyl- and propyl- tiglate) were only produced by the S0 strain, whereas 3-methylbututyl (E)-3-methylbut-2-enoate (isoamyl 3-meyhylcrotonate) and ethyl (E)-hex-2-enoate were produced by two strains isolated from South African fauna but not by S0. Indeed, 3-methylbutyl (E)-3-methylbut-2-enoate was detected in the volatilome of S12, whereas ethyl (E)-hex-2-enoate was produced by the S12 and S91 strains, both isolated from feces collected at Lory Park from the black-footed cat *(Felis nigripes)* and serval *(Leptailurus serval*), respectively. As far as we know, 3-methylbutyl (E)-3-methylbut-2-enoate has never been reported in the volatilome of yeast strains and its flavor has not been described yet. Similarly, the occurrence of ethyl (E)-hex-2-enoate (known for its fruity-like aroma) seems to be very anecdotal. Indeed, nowadays, only one study [16] has mentioned the production of ethyl (E)-hex-2-enoate by the non-conventional yeast *Geotrichum marinum* when fermenting YPD medium.

### 3.4. Multivariate Analysis of ILV^+^ Aroma-Producing Yeasts, Isolated Based on Their Origins and Their Volatile Organic Compound Production

To better visualize how strains behave with respect to their performance to produce VOCs (number of total VOCs, number of acids, number of alcohols, number of esters and number of α-unsaturated esters) and their origin (feces from carnivorous and herbivorous mammals, from reptiles and from domestic and natural animal habitats), a hierarchical cluster analysis (HCA) was carried out on the 22 isolated ILV^+^ yeast isolates.

**Figure 1:**
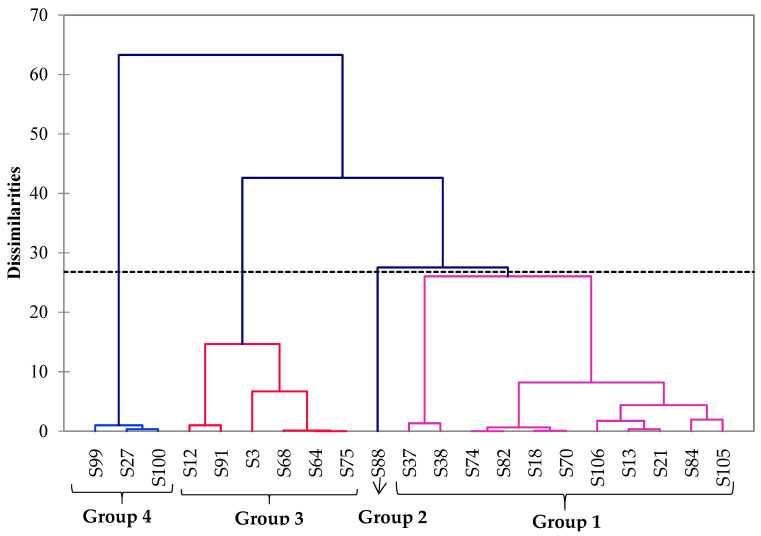
Cluster analysis of the isolated strains producing VOCs. Strains were grouped based on their flavor production and their origin using Ward’s method, performed using XL Stat Applied Sensory software (2020.1.3) Automatic truncation (dotted line) allowed the identification of four consistent groups of strains. Most of the strains were classified into group 1 (11 strains) and group 3 (6 strains). Group 4 included three strains. Strain S88 was assigned to group 2. The dendrogram is more flattened for group 4, suggesting that this group of strains is more homogeneous than the two other groups.

The dendrogram obtained *via* HCA (Figure 1) suggests that the strains could be classified into four significant groups. Group 1 contained yeasts that are commonly characterized by the production of a low amount and diversity of VOCs. Strain S106 was assigned to this group, although it produced 15 different esters including 3 α-unsaturated esters. Group 2 encompassed only strain S88, likely because it only produced three esters (Table 3). Group 3 contained strains isolated from the feces of carnivorous animals living in natural or domestic habitats and was characterized by the production of a higher amount and greater diversity of VOCs, including esters and α-unsaturated esters. In this group, the S3 strain appeared to be the most divergent strain as it produced a lower number of VOCs compared to the others. On the other hand, this group is likely the most interesting as it shows higher diversity in α-unsaturated esters. Finally, group 4 contained three isolates characterized by the production of small amounts of the different VOCs. The isolates of this group mainly differed from those from group 3 because they were isolated from herbivorous animals (vs. carnivorous animals for group 3) living in natural habitats (Moreleta Kloof Nature Reserve).

**Figure 2:**
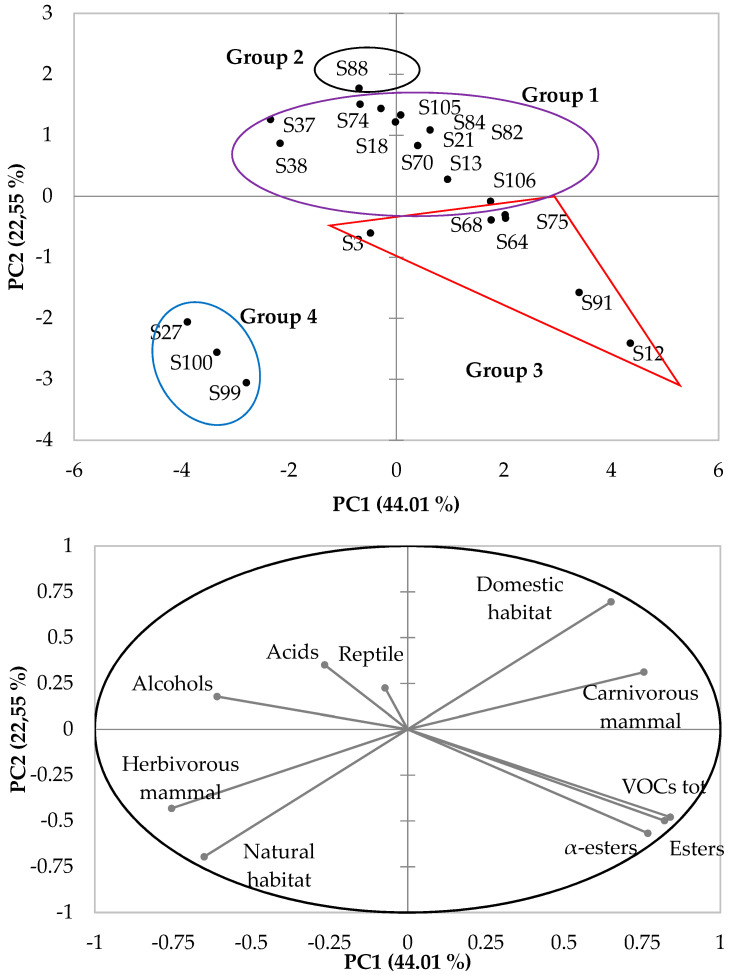
Score plot of PC2 versus PC1 according to strains’ flavor production and their origins. Component analysis was performed using XL Stat Applied Sensory software (2020.1.3). Group 1 included S13, S18, S21, S37, S38, S70, S74, S82, S84, S105 and S106. Group 2 was only composed of S88. Group 3 included S3, S12, S64, S68, S75 and S91. Group 4 included S27, S99 and S100.

In addition to the HCA, principal component analysis (PCA) was constructed on the basis of the same parameters. The Pearson correlation matrix obtained for this PCA (Figure 2, Table 5) shows that the number of acids was the only parameter that did not correlate with any of the other parameters, probably because they represented only a few parts of the VOCs detected, compared to the other categories of volatiles. The total number of VOCs was strongly correlated with the number of esters (Pearson correlation r of 0.990) and the number of α-unsaturated esters (r = 0.938), but negatively correlated with the number of alcohols (only 1 alcohol found, which corresponded to 2-phenylethanol, r = −0.514). These results confirm the activity of the EP for strains producing VOCs and the occurrence of an effective esterase for these strains. Moreover, they tend to demonstrate that ILV^+^ isolates exhibit active β-oxidation of the BCCA pathway. Furthermore, the number of esters and the number of α-unsaturated esters were highly correlated with each other (r = 0.903) confirming that these strains display effective esterases, which are able to metabolize esterified alcohol and acids from the EP, but also the enoyl-CoA intermediate from the β-oxidation of the BCCA pathway.

### 3.5. Identification of Strains S12 and S91

In the volatilome of strains that belong to group 3, it was interestingly noticed that the S12 and S91 strains produced two novel α-unsaturated esters, namely 3-methylbutyl (E)-3-methylbut-2-enoate and ethyl (E)-hex-2-enoate, which were not found in the aroma bouquet in *S. suaveolens*. The molecular identification of these two strains was performed *via* amplification and sequencing of their ribosomal Internal Transcribed Spacer (ITS) region (non-coding and species-specific domains) (See Supplementary Material Table S1). The ITS sequences of S12 and S91 were deposited to the GenBank database with the accession numbers of “OM397075” and “OM397076”, respectively. Blast analysis (NCBI) of these sequenced DNA regions indicated that they are two identical strains and they displayed 100% sequence identity with the yeast *Galactomyces candidus* (a teleomorph of *Geotrichum candidum*, NCBI accession number: MK381259.1). These results were supported by carrying out sequence alignment using the MycoBank reference database (See Supplementary Materials Tables S2 and S3). This result was consistent with the overall results of the semi-quantitative analysis of VOCs produced by strains S12 and S91, as both displayed similar overall production of VOCs (≈4.9 mg·L^−1^), esters (≈4.0 mg·L^−1^) and α-unsaturated esters (≈0.6 mg·L^−1^). Data from the literature have already mentioned the production of α-unsaturated esters by *Galactomyces candidus* [17,19], but none of them have reported the production of 3-methylbutyl (E)-3-methylbut-2-enoate and ethyl (E)hex-2-enoate. In this study, semi-quantitative analysis of VOCs showed that these compounds were produced at very low concentrations by strain S12 (≈3 µg·L^−1^ and 12 µg·L^−1^, respectively), but surprisingly, the production of ethyl (E)-hex-2-enoate by S91 reached 110 µg·L^−1^. Thus, although the molecular data did not allow us to distinguish the S12 strain from S91, their respective different volatilomes tend to suggest that they could belong to different subspecies, as can be observed on the dendrogram obtained *via* HCA (Figure 1). To confirm this hypothesis, another molecular marker for the detection of intra-species polymorphisms would be needed.

## 4. Conclusions

This work aimed to isolate new strains producing α-unsaturated esters using an original method based on the metabolism of *S. suaveolens*, taken as model due to its large variety of α-unsaturated ester production. Therefore, yeast strains were isolated from the feces of South African fauna, and 43 of them were selected for their ability to grow on branched-chain amino acids (isoleucine, leucine and valine) as a sole carbon source (ILV^+^ phenotype). Twelve α-unsaturated ester-producing yeast strains were thus isolated, among which two strains, both identified as *Galactomyces candidus* (*Geotrichum candidum*), produced two novel α-unsaturated esters that were not identified in the volatilome of the model strain, *S. suaveolens*. The results of this study demonstrated the efficiency of this robust method to screen for novel α-unsaturated ester-producing yeast strains. Moreover, they prompted an interest in broadening the search for aroma-producing non-*Saccharomyces* yeasts from original ecological niches such as animals’ feces. However, this study did not allow us to provide evidence of a strict correlation between the ILV^+^ phenotype and the production of α-unsaturated esters. A better understanding of the role and the production of α-unsaturated esters by yeasts is therefore necessary to improve the screening method described in this work.

## Figures and Tables

**Table 1:** List of wild animal feces collection sites in South Africa.

Site Name	Type of Site and Address
Moreleta Kloof Nature Reserve	Game reserve (Helios Street, Moreleta park, Pretoria, Gauteng)
Kruger National Park	National Park (Kruger National Park, Mpumalanga)
Lory Park Zoo	Zoo (180/1 Kruger Road, President Park, Midrand, Gauteng)
McCrindle Farm	Game reserve (Pretoria, Gauteng)
Emoya big cat sanctuary	Zoo (Farm Milumbe, D1939, Lephalale, Limpopo)
Elephant Sanctuary	Zoo (Hartbeespoort dam)
Moholoholo Animal Rehabilitation center	Zoo (Limpopo)

**Table 2:** Number of different strains originating from diverse South African animal feces and isolated on YPD-chloramphenicol medium.

Animals	Type of Animal	Sampling	Origin	No. of Different Strains
Aardwolf *(Proteles cristata)*	Carnivorous mammal	Freeze-dried	Not communicated	2 (S1/S2)
Abyssinian hornbill (*Bucorvus abyssinicus*)	Bird	Swab	Lory Park Zoo	0
Bateleur eagle *(Terathopius ecaudatus)*	Raptor	Swab	Moholoholo Animal Rehabilitation Center	0
Black-and-white-casqued hornbill *(Bycanistes subcylindricus)*	Bird	Swab	Lory Park Zoo	1 (S31)
Black-crested gibbon *(Nomascus concolor*)	Herbivorous mammal	Swab	Lory Park Zoo	2 (S3 */S4 *)
Black jaguar *(Panthera pardus)*	Carnivorous mammal	Swab	Lory Park Zoo	3 (S6/S7/S8)
Black-footed cat *(Felis nigripes)*	Carnivorous mammal	Tubes	Lory Park Zoo	4 (S12 */S14/S15/S16))
		Swab	Lory Park Zoo	3 (S13 */S17 */S18 *)
Blesbok *(Damaliscus pygarus*)	Herbivorous mammal	Tube	Moreleta Kloof Nature Reserve	2 (S9/S10)
Brown lemur *(Eulemur fulvus)*	Herbivorous mammal	Swab	Lory Park Zoo	3 (S58 */S59/S60 *)
Brown jaguar *(Panthera onca*)	Carnivorous mammal	Swab	Lory Park Zoo	5 (S20/S21 */S22 */S23/S24)
Buffalo *(Syncerus caffer*)	Herbivorous mammal	Swab	Kruger National Park	2 (S28 */S29 *)
		Freeze-dried	Not communicated	1 (S30)
Bushbuck (*Tragelaphus sylvaticus*)	Herbivorous mammal	Tube	Moreleta Kloof Nature Reserve	2 (S26/S27 *)
		Swab	Moreleta Kloof Nature Reserve	0
Cape vulture (*Gyps coprotheres*)	Raptor	Swab	Moholoholo Animal Rehabilitation Center	0
Cheetah *(Acinonyx jubatus*)	Carnivorous mammal	Tube	Lory Park Zoo	2 (S32/S33 *)
		Swab	Lory Park Zoo	2 (S34/S35)
		Freeze-dried	Not communicated	0
Duiker (*Sylvicapra grimmia)*	Herbivorous mammal	Tube	Moholoholo Animal Rehabilitation Center	2 (S37 */S38 *)
Elephant *(Loxodonta africana)*	Herbivorous mammal	Swab	Kruger National Park	0
		Freeze-dried	Not communicated	1 (S42)
		Swab	Elephant Sanctuary	3 (S39/S40 */S41)
Gibbon (*Hylobates lar)*	Herbivorous mammal	Swab	Lory Park Zoo	3 (S43/S44 */S45)
Giraffe *(Giraffa cameopardalis*)	Herbivorous mammal	Freeze-dried	Not communicated	1 (S46)
Hyena *(Crocuta crocuta*)	Carnivorous mammal	Swab	Lory Park Zoo	1 (S47)
		Freeze-dried	Not communicated	1 (S48)
Impala *(Aepyceros melampus*)	Herbivorous mammal	Tube	Kruger National Park	2 (S49/S50)
		Tube	McCrindle Farm	2 (S51/S52)
King vulture *(Sarcoramphus papa)*	Raptor	Swab	Lory Park Zoo	0
Kudu (*Tragelaphus strepsiceros)*	Herbivorous mammal	Tube	McCrindle Farm	2 (S53/S54)
Lappet-faced vulture *(Torgos tracheliotos)*	Raptor	Swab	Moholoholo Animal Rehabilitation Center	0
Lemur *(Varecia variegate*)	Herbivorous mammal	Tube	Lory Park Zoo	3 (S55/S56/S57)
		Swab	Lory Park Zoo	0
Leopard *(Panthera pardus*)	Carnivorous mammal	Swab	Lory Park Zoo	7 (S62 */S63 */S64 */S65 */S68 */S69/S70 *)
		Tube	Lory Park Zoo	2 (S66/S67)
		Freeze-dried	Not communicated	0
Lion *(Panthera leo*)	Carnivorous mammal	Freeze-dried	Not communicated	1 (S80)
		Tube	Emoya big cat sanctuary	10 (S71/S72 */S78 */S73/S74 */S75 */S76/S77/S79/S80)
		Swab	Emoya big cat sanctuary	1 (S78)
Long-tailed hornbill *(Tropicranus albocristatus)*	Raptor	Swab	Lory Park Zoo	0
Meerkat *(Suricata suricatta*)	Carnivorous mammal	Tube	Lory Park Zoo	3 (S82 */S83/S84 *)
Nile crocodile *(Crocodylus niloticus*)	Reptile	Swab	Lory Park Zoo	4 (S85/S86/S87/S88 *)
Ostrich *(Struthio camelus*)	Bird	Swab	Moreleta Kloof Nature Reserve	2 (S89/S90)
Palm nut vulture *(Gypohierax angolensis)*	Raptor	Swab	Lory Park Zoo	0
Serval *(Leptailruus serval*)	Carnivorous mammal	Tube	Lory Park Zoo	3 (S91 */S92 * /S93)
		Swab	Lory Park Zoo	2 (S94/S95)
Southern hornbill *(Bucorvus leadbeateri*)	Raptor	Swab	Lory Park Zoo	0
Springbok *(Antidorcas marsupialis*)	Herbivorous mammal	Tube	Moreleta Kloof Nature Reserve	6 (S96/S97 */S98/S99 */S100 */S101)
Tawney eagle (*Aquila rapax)*	Raptor	Swab	Moholoholo Animal Rehabilitation Center	2 (S102/S103)
Tiger *(Panthera tigris*)	Carnivorous mammal	Tube	Lory Park Zoo	4 (S104/S105 */S106 */S108/ S109)
		Swab	Lory Park Zoo	1 (S107)
White-headed vulture *(Trigonoceps occipitalis)*	Raptor	Swab	Lory Park Zoo	3 (S114 /S115/S116)
		Swab	Moholoholo Animal Rehabilitation Center	0
White lion *(Panthero leo*)	Carnivorous mammal	Swab	Lory Park Zoo	2 (S117 */S118)
White rhino (*Ceratotherium simum)*	Herbivorous mammal	Freeze-dried	Not communicated	2 (S119/S120)
Wild dog *(Lycaon pictus*)	Carnivorous mammal	Freeze-dried	Not communicated	1 (S113)
Wildebeest *(Connochaetes gnou*)	Herbivorous mammal	Tube	Moreleta Kloof Nature Reserve	2 (S111/S112)
		Freeze-dried	Not communicated	1 (S110)
Zebra *(Equus zebra*)	Herbivorous mammal	Swab	Moreleta Kloof Nature Reserve	2 (S121 */S122)
		Tube	Moreleta Kloof Nature Reserve	2 (S123 */S124 *)
		Swab	McCrindle Farm	1 (S125 *)
			**TOTAL**	**119**

Collection was performed using pre-labeled sterile swabs or tubes, which were hermetically sealed and transported from the site of sampling to the laboratory. Freeze-dried samples were obtained from an anonymous donator. The star (*) designates isolates whose phenotype is of the ILV^+^ type, i.e., capable of developing on media containing only branched-chain amino acids (isoleucine, leucine and valine) as a source of carbon.

**Table 3:** Volatile organic compounds produced by the isolated strain during growth on yeast–peptone–dextrose agar medium.

Volatile Compounds	Odor Type ^(1)^	IRR_exp_^(2)^	IRR_th_^(3)^	Producing Strains
**ACIDS**				
2-methylbutanoic acid	Over-ripe fruit, cashew, sweet	856	846	S38
3-methylbutanoic acid	Sweet	837	828	S37; S84; S105
Butanoic acid	Rancid	792	780	S37
**ALCOHOL**				
2-phenylethanol	Flower, honey, rose	1101	1114	S0; S13; S18; S21; S27; S37; S38; S70; S74; S82; S99; S100; S105; S106
**ESTERS**				
2-methylbutyl 2-methylbutanoate	Fruit, apple, rum, berry	1110	1104	S0
2-methylbutyl 3-methylbutanoate	Apple, cheese, earth	1112	1107	S0
2-methylbutyl butanoate	Fruit, spice, butter	1057	1056	S0
2-methylbutyl propanoate	Sweet, banana, fruit, apple, melon,	975	975	S0
2-methylpropyl 2-methylbutanoate	Sweet, fruit	1002	1004	S0
2-methylpropyl 2-methylpropanoate	Pineapple, grape skin, tropical	955 ^(4)^	909	S0; S91; S106
2-methylpropyl 3-methylbutanoate	Fruit, apple, raspberry	892	904	S0; S12; S13; S18; S68; S75; S99
2-methylpropyl butanoate	Sweet, fruit	959	955	S0
2-methylpropyl propanoate	Fruit, green ether, sweet, banana	869	863	S12
2-phenylethyl 2-methylbutanoate	Sweet, fruit, herb, floral	1494	1493	S0
2-phenylethyl 3-methylbutanoate	Apricot, sweet, bitter	1486	1488	S0; S12; S13; S27; S75
2-phenylethyl acetate	Rose, floral, fruit, sweet	1261	1265	S0
3-methylbutyl 2-methylbutanoate	Fruit	1093	1102	S0; S12; S13; S99
3-methylbutyl 2-methylpropanoate	Mixed fruit	1011	1013	S0; S12; S75
3-methylbutyl 3-methylbutanoate	Sweet, apple, fruit	1099	1103	S12; S13; S18; S75; S99; S105;
3-methylbutyl acetate	Pear, banana	872	871	S0; S3; S12; S18; S21; S64; S68; S70; S75; S88; S91; S106
3-methylbutyl pentanoate	Ripe apple	1097	1090	S0; S3; S64
3-methylbutyl propanoate	Apricot, pineapple	968	964	S0; S12; S68; S75; S91; S106
Butyl 2-methylbutanoate	Fresh, sweet, fruit	1039	1043	S0; S12; S91;
Butyl 2-methylpropanoate	Fruit	951	955	S0; S12; S64; S68; S91; S106
Butyl 3-methylbutanoate	Apple, pear, sweet, pineapple, green	1043	1047	S0; S12; S106
Butyl butanoate	Fresh, sweet, fruit	995	993	S12; S21; S64; S91; S100; S106
Butyl propanoate	Sweet, fruit, rum	907	910	S12; S64; S75; S91; S106
Ethyl 2-methylbutanoate	Fruit, green, apple, flower	842	846	S0; S3; S12; S18; S21; S68; S75; S91; S99; S105; S106
Ethyl 3-methylbutanoate	Fruit, blueberry	847	849	S0; S3; S12; S13; S18; S21; S64; S68; S75; S91; S99; S105; S106
Ethyl butanoate	Fruit, pear, pineapple	799	800	S0; S3; S12; S21; S37; S38; S64; S68; S70; S75; S84; S88; S91; S100; S105; S106
Ethyl hexanoate	Fruit, strawberry, anise	999	996	S12; S64; S68; S84; S91
Ethyl octanoate	Flower, fruit, menthol, anise, sweet	1196	1198	S0; S91
Ethyl pentanoate	Fruit, orange, green	890	898	S12; S13; S64; S68; S75; S84; S91; S106
Octyl 2-methylpropanoate ^(4)^	Cream, wax, fruit, earth, fat	1349	1394	S0
Octyl 2-methylbutanoate	Green, must, fruit	1432	1438	S0
Octyl 3-methylbutanoate	Rose, honey, apple, pineapple	1436	1440	S0
Octyl acetate	Floral, fruit, sweet	1115	1149	S0
Octyl butanoate	Herb, fruit, green	1391	1434	S0
Pentyl butanoate	Apricot, pineapple	1052	1093	S12; S91
Propyl 3-methylbutanoate	Sweet, fruit	947	943	S0; S12; S91
**α-UNSATURATED ESTERS**				
2-methylpropyl (E)-2-methylbut-2-enoate	Herb, pungent	1039	1034	S0; S12; S13; S91
3-methylbutyl (E)-2-methylbut-2-enoate	Floral	1190	1168	S0; S12; S91
3-methylbutyl (E)-3-methylbut-2-enoate		1178	1184	S12
Butyl (E)-2-methylbut-2-enoate	Floral, herb, warm, fruit	1091	1068	S0
Ethyl (E)-2-methylbut-2-enoate	Fruit	938	936	S0; S3; S12; S13; S64; S68; S75; S84 S91; S99; S100; S105; S106
Ethyl (E)-3-methylbut-2-enoate	-	922	911	S0; S3; S12; S13; S64; S68; S75; S84; S91; S99; S105; S106
Ethyl (E)-but-2-enoate	Fruit, caramel, pungent	835	833	S0; S21; S64; S106
Ethyl (E)-hex-2-enoate	Fruit, slight, pungent	1041	1025	S12; S91
Propyl (E)-2-methylbut-2-enoate	-	1039	1034	S0

^(1)^ Odor types were derived from “Flavornet and human odor space” and the “Database of pheromones and semiochemicals”. ^(2)^ Experimental incidence rate ratios (IRR_exp_) were calculated according to Kovats (1958) based on experimental retention time [49]. ^(3)^ Theorical incidence rate ratios (IRR_th_) were derived from Nagata, 2003; Chen et al., 2006; Czerny et al., 2008; and “Odor and flavor detection thresholds in water (in parts per billion)” from Leffingwell and Associates [50,51,52,53]. ^(4)^ Tentative.

**Table 4:** Classification of the VOCs produced by each isolated strain.

Strains	VOC Tot.	Acids	Alcohols	Esters	α-Esters
**S0**	38	-	1	30	7
**S3**	7	-	-	5	2
**S12**	27	-	-	21	6
**S13**	10	-	1	6	3
**S18**	6	-	1	5	-
**S21**	8	-	1	6	1
**S27**	2	-	1	1	-
**S37**	5	2	1	2	-
**S38**	4	1	1	2	-
**S64**	13	-	-	10	3
**S68**	12	-	-	10	2
**S70**	4	-	1	3	-
**S74**	1	-	1	-	-
**S75**	14	-	-	12	2
**S82**	1	-	1	-	-
**S84**	6	1	-	3	2
**S88**	3	-	-	3	-
**S91**	21	-	-	16	5
**S99**	8	-	1	5	2
**S100**	5	-	1	3	1
**S105**	8	1	1	5	1
**S106**	16	-	1	12	3

VOC Tot.—number of different VOCs; Acids—number of different acids; Alcohols—number of different alcohols; Esters—number of different esters; α-esters—number of different α-unsaturated esters.

**Table 5:** Pearson (n) correlation matrix obtained for principal component analysis.

Variable	VOC	Ac.	Alc.	Est.	α-Est.	Herb.	Carn.	Rept.	Nat.	Dom.
**VOCs**	**1**									
**Ac.**	−0.211	**1**								
**Alc.**	**−0.514**	0.169	**1**							
**Est.**	**0.990**	−0.288	**−0.560**	**1**						
**α-est.**	**0.938**	−0.262	**−0.551**	**0.903**	**1**					
**Herb.**	−0.336	0.315	0.279	−0.378	−0.278	**1**				
**Carn.**	0.410	−0.256	−0.139	0.423	0.361	**−0.894**	**1**			
**Rept.**	−0.194	−0.101	−0.285	−0.134	−0.209	−0.141	−0.316	**1**		
**Nat.**	−0.228	−0.185	0.320	−0.244	−0.139	**0.645**	**−0.577**	−0.091	**1**	
**Dom.**	0.228	0.185	−0.320	0.244	0.139	**−0.645**	**0.577**	0.091	**−1.000**	**1**

Bold values are significantly different from 0 at a significance level of α = 0.05. VOCs—number of different VOCs; Ac.—number of different acids; Alc.—number of different alcohols; Est.—number of different esters; α-est.—number of different α-unsaturated esters; Herb—herbivorous mammal; Carn.—carnivorous mammal; Rept.—reptile; Nat.—natural habitat; and Dom.—domestic habitat origin of feces from which strains have been isolated.

## Data Availability

Not applicable.

## Supplementary Materials

The following supporting information can be downloaded at: https://www.mdpi.com/article/10.3390/life12121999/s1, Table S1: Identification of strains S12 and S91 including details from sequencing results of ITS and D1/D2 domains; Table S2. Alignment results of ITS sequences of strains S12 and S91 in the GenBank database; Table S3. Alignment results of ITS sequences of strains S12 and S91 in the MycoBank database.
