# Supplementary material for: Screening for Volatile α-Unsaturated Ester-Producing Yeasts from the Feces of Wild Animals in South Africa
Source: Life (Basel). 2022 Nov 30;12(12):1999. doi: 10.3390/life12121999 (PMC9782132; doi:10.3390/life12121999)
Supplement: Supplementary file 1 [file life-12-01999-s001.zip › life-1947407-supplementary.pdf]

Supplementary Material

Supplementary Material Table S1. ITS Sequencing results of strains S12 and S91

	**ITS Sequences**
**S12**	AATTATACAGCAAACAATAATTTTATAGTCAAAACAAAAAAAAAATTAAAACTTTTAACAATGGATCTCTTGGTTCTCGTATCGATGAAGAACGCAGCGAAACGCGATATTTCTTGTGA ATTGCAGAAGTGAATCATCAGTTTTTGAACGCACATTGCACTTTGGGGTATCCCCCAAAGTATACTTGTTTGAGCGTTGTTTCTCTCTTGGAATTGCATTGCTTTTCTAAAATATTGAATC AAATTCGTTTGAAAAACAACACTATTCAACCTCAGATCAAG
**S91**	AATTAATTGTGAAAATTATACAGCAAACAATAATTTTATAGTCAAAACAAAAAAAAAATTAAAACTTTTAACAATGGATCTCTTGGT

Supplementary Material Table S2. Alignment results of ITS sequences of strains S12 and S91 in the GenBank database

	**Identification**	**#NCBI**	**% homology**	**strand**	**Aligned model sequences**
**S12**	*Galactomyces candidus**	MK381259.1	100%	(+/+)	GAAAATTATACAGCAAACAATAATTTTATAGTCAAAACAAAAAAAAAATTAAAACTTTTAACAATGGATCTC
**S91**	*Galactomyces candidus**	MK381259.1	100%	(+/+)	AATTAATTGTGAAAATTATACAGCAAACAATAATTTTATAGTCAAAACAAAAAAAAAATTAAAACTTTTAACAATGGA TCTCTTGGT

**Galactomyces candidus* is a synonymous of *Dipodascus geotrichum* and of *Geotrichum candidum* (Mycologie, University of Adelaide - https://www.adelaide.edu.au/mycology/fungal-descriptions-and-antifungal-susceptibility/hyphomycetes-conidial-moulds/geotrichum-candidum)

Supplementary Material Table S3. Alignment results of ITS sequences of strains S12 and S91 in the MycoBank database

	**Identification**	**#MycoBank**	**% homology**	**strand**	**Aligned model sequences**
**S12**	*Dipodascus geotrichum**	KP769540	100%	(+/+)	AATTATACAGCAAACAATAATTTTATAGTCAAAACAAAAAAAAAATTAAAACTTTTAACAATGGATCTCTTGGTTCTCG TATCGATGAAGAACGCAGCGAAACGCGATATTTCTTGTGAATTGCAGAAGTGAATCATCAGTTTTTGAACGCACATTGC ACTTTGGGGTATCCCCCAAAGTATACTTGTTTGAGCGTTGTTTCTCTCTTGGAATTGCATTGCTTTTCTAAAATATTGAAT CAAATTCGTTTGAAAAACAACACTATTCAACCTCAGATCAAG
**S91**	*Dipodascus geotrichum **	KP769540	100%	(+/+)	AATTAATTGTGAAAATTATACAGCAAACAATAATTTTATAGTCAAAACAAAAAAAAAATTAAAACTTTTAACAATGGA TCTCTTGGT

**Dipodascus geotrichum* is a synonymous of *Galactomyces candidus* and of *Geotrichum candidum* (Mycologie, University of Adelaide - https://www.adelaide.edu.au/mycology/fungal-descriptions-and-antifungal-susceptibility/hyphomycetes-conidial-moulds/geotrichum-candidum)
